# Evaluation of dyspnea of unknown etiology in HIV patients with cardiopulmonary exercise testing and cardiovascular magnetic resonance imaging

**DOI:** 10.1186/s12968-020-00664-6

**Published:** 2020-10-12

**Authors:** Andrew J. Patterson, Anuja Sarode, Sadeer Al-Kindi, Lauren Shaver, Rahul Thomas, Evelyn Watson, Mohamad Amer Alaiti, Yuchi Liu, Jessie Hamilton, Nicole Seiberlich, Imran Rashid, Robert Gilkeson, Robert Schilz, Brian Hoit, Trevor Jenkins, Melissa Zullo, Eduardo Bossone, Christopher Longenecker, Orlando Simonetti, Sanjay Rajagopalan

**Affiliations:** 1 Harrington Heart and Vascular Institute, University Hospitals, Cleveland, OH USA; 2 Kent State University, College of Public Health, Kent, OH USA; 3 Michigan University, Department of Biomedical Engineering, Ann Arbor, MI USA; 4 University Hospitals Cleveland Medical Center, Department of Radiology, Cleveland, OH USA; 5 University Hospitals Cleveland Medical Center, Department of Pulmonology, Cleveland, OH USA; 6 Cardiology Division, Cardarelli Hospital, Naples, Italy; 7 Ohio State University Department of Cardiovascular Medicine, Columbus, OH USA

**Keywords:** HIV, CPET, Exercise CMR, Contractile Reserve

## Abstract

**Aim:**

Human Immunodeficiency Virus **(**HIV) patients commonly experience dyspnea for which an immediate cause may not be always apparent. In this prospective cohort study of HIV patients with exercise limitation, we use cardiopulmonary exercise testing (CPET) coupled with exercise cardiovascular magnetic resonance (CMR) to elucidate etiologies of dyspnea.

**Methods and results:**

Thirty-four HIV patients on antiretroviral therapy with dyspnea and exercise limitation (49.7 years, 65% male, mean absolute CD4 count 700) underwent comprehensive evaluation with combined rest and maximal exercise treadmill CMR and CPET. The overall mean oxygen consumption (VO_2_) peak was reduced at 23.2 ± 6.9 ml/kg/min with 20 patients (58.8% of overall cohort) achieving a respiratory exchange ratio > 1. The ventilatory efficiency (VE)/VCO_2_ slope was elevated at 36 ± 7.92, while ventilatory reserve (VE: maximal voluntary ventilation (MVV)) was within normal limits. The mean absolute right ventricular (RV) and left ventricular (LV) contractile reserves were preserved at 9.0% ± 11.2 and 9.4% ± 9.4, respectively. The average resting and post-exercise mean average pulmonary artery velocities were 12.2 ± 3.9 cm/s and 18.9 ± 8.3 respectively, which suggested lack of exercise induced pulmonary artery hypertension (PAH). LV but not RV delayed enhancement were identified in five patients. Correlation analysis found no relationship between peak VO_2_ measures of contractile RV or LV reserve, but LV and RV stroke volume correlated with PET CO_2_ (*p* = 0.02, *p* = 0.03).

**Conclusion:**

Well treated patients with HIV appear to have conserved RV and LV function, contractile reserve and no evidence of exercise induced PAH. However, we found evidence of impaired ventilation suggesting a non-cardiopulmonary etiology for dyspnea.

## Introduction

Unexplained dyspnea in patients with human immunodeficiency virus (HIV) can be multifactorial and may portend a poor prognosis especially with diagnoses such as HIV pulmonary arterial (PA) hypertension (PAH) [1–3]. Indeed, the earliest signs of eventual PAH are impaired right ventricular (RV) contractile reserve and a phase of exercise induced PAH [4] [5]. The presence of these features may help identify subsets of patients with propensity for developing resting PAH and/or RV contractile dysfunction for preemptive therapy [6]. While the gold standard for evaluation of cardiopulmonary performance and assessment of PA pressure remains invasive right heart catheterization in combination with exercise, the use of such an approach for comprehensive screening is fairly involved [7, 8]. Other tools such as stress echocardiography to evaluate for changes in tricuspid valve velocities, increase in E/e’ and contractile dysfunction are often used, but are limited by imaging windows that could be suboptimal in many patients, particularly with exercise [8]. Coupling cardiovascular magnetic resonance (CMR) with cardiopulmonary exercise testing (CPET) may allow comprehensive noninvasive functional and structural analysis of patients with dyspnea of unclear etiology and could provide valuable prognostic and diagnostic information [9]. In this prospective study, we evaluated HIV patients with subjective exercise limitation, with no other obvious evidence of cardiopulmonary disease to comprehensively assess ventricular contractile reserve, PA velocities and cardiopulmonary ventilatory indices using a treadmill exercise CMR imaging protocol combined with CPET.

## Methods

### Patient selection

**Fig. 1:**
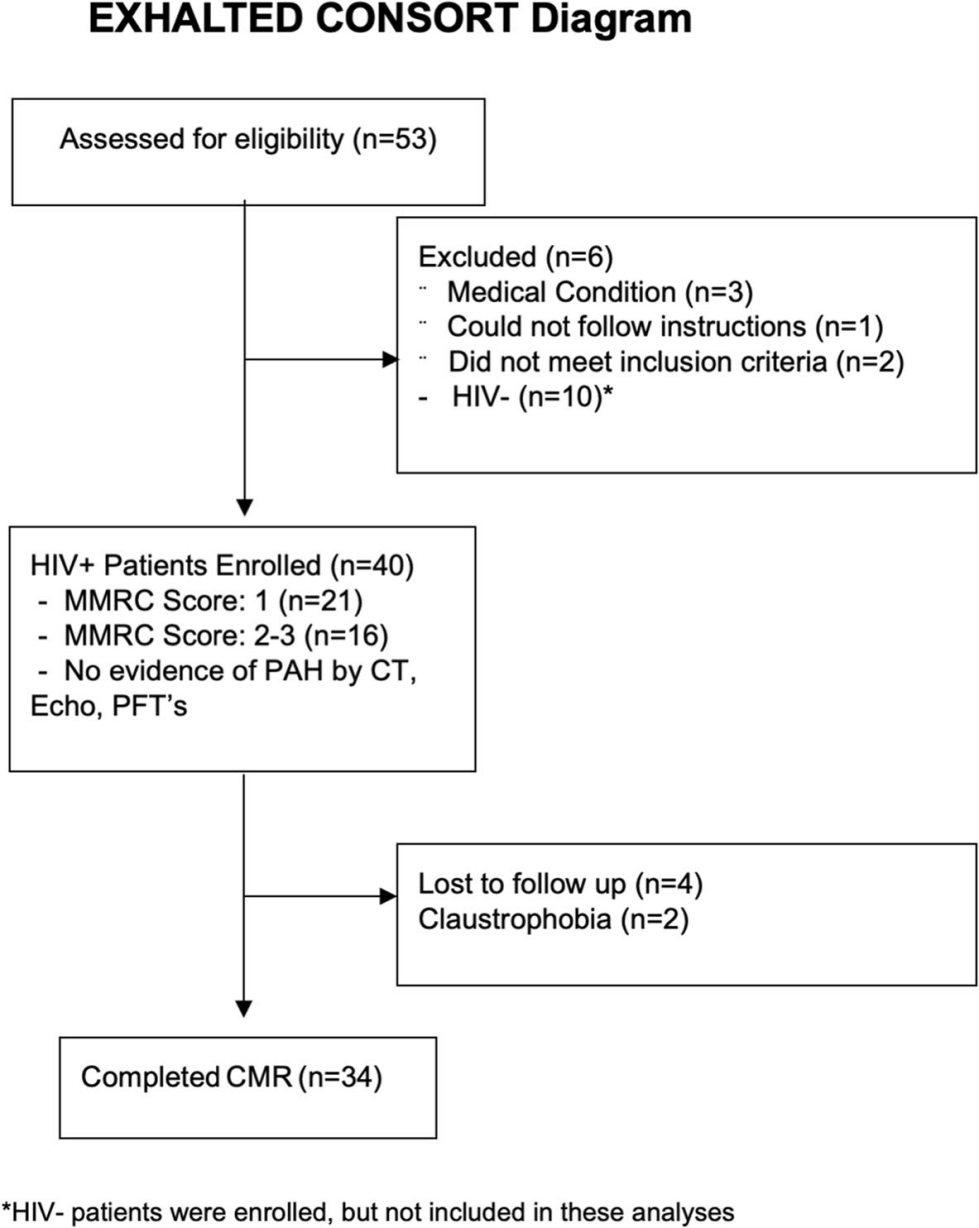
Diagram of selected patients that underwent combined CPET/CMR for evaluation of dyspnea. A total of 53 enrolled of which 34 completed screening and underwent testing. CPET – cardiopulmonary exercise testing, CT - computed tomography, CMR – cardiovascular magnetic resonance, HIV - human immunodeficiency virus, MMRC - Modified Medical Research Council, PFTs - pulmonary function testing

A total of 34 patients were enrolled out of 53 eligible patients with HIV that were referred for functional assessment between May 2017 and January 2019 (Fig. 1). All patients signed a consent form and the study was conducted in accordance with IRB standards of University Hospitals Cleveland Medical Center and in accordance with Helsinki convention.

The inclusion criteria included adults aged 18–65 years with confirmed HIV, with unexplained persistent or progressively worsened dyspnea for at least 6 weeks, and were described previously [10]. Screening echocardiography with a cutoff of resting PA systolic pressures of ≤40 mmHg using peak tricuspid regurgitation velocities coupled with right atrial pressures, pulmonary function testing to rule out obstructive and restrictive pulmonary disease, computed tomography (CT) screening if indicated to exclude parenchymal pulmonary disease as well as evidence of thromboembolic disease with main PA size (< 3 cm) were used to assess for eligibility. Patients were required to have exercise limitation based on the Modified Medical Research Council (MMRC) dyspnea scale, the most commonly used validated subjective scale to assess shortness of breath in patients with respiratory disease as previously described [11]. The scale uses a 0–4 scale with 0 indicating shortness of breath only with strenous exercise. 1–3 are graded progressive symptoms. Four indicates the subject is too short of breath to leave their house or they have shortness of breath when dressing.

Exclusion criteria included contraindications to CMR (e.g., metallic implants, severe claustrophobia); acute coronary syndrome, transient ischemic attack, stroke or critical limb ischemia during the last 6 months or coronary/peripheral revascularization within the last 3 months; concurrent potentially life threatening arrhythmia or symptomatic arrhythmia; documented left ventricular (LV) ejection fraction (LVEF) < 45%; evidence of pulmonary infection in the past 4 weeks; Evidence of another etiology of individual’s dyspnea such as reactive airway disease, heart failure, intrinsic lung disease, and decompensated cirrhosis; chronic kidney disease with estimated glomerular filtration rate (eGFR) of < 40 ml/min/1.73 m^2^ or recent acute kidney injury; hemoglobin < 8 g/dL; platelets < 50,000/mm; pregnancy; active drug use, either by history or urine toxicology screen.

### Stress protocol

**Fig. 2:**
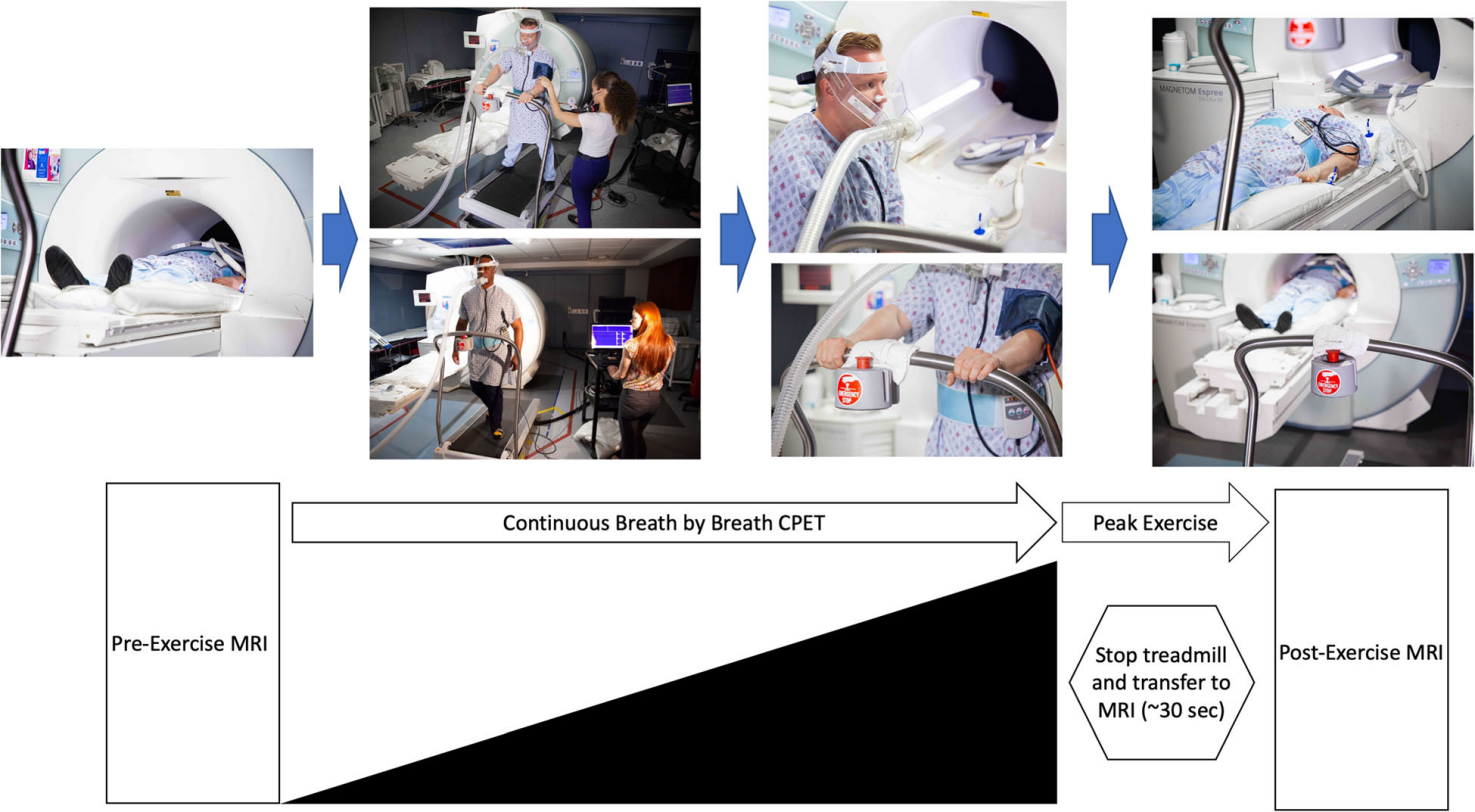
Central Illustration. Flow diagram with matching photos depicting the sequence of events for stress treadmill CMR-CPET protocol. Pre exercise imagines were obtained on a 1.5 T CMR system. Subjects exercised on a CMR compatible hydraulic treadmill with simultaneous measurement of ventilation with CPET. Immediately following cessation of exercise, subjects were transferred back to the CMR station for post exercise images

Subjects were scheduled for combined CPET and exercise CMR (Central Illustration: Fig. 2) as previously described [10]. Pre-exercise images were reviewed by an experienced cardiologist and/or radiologist prior to exercise. After the completion of pre-exercise images, patients were transferred to a CMR-compatible treadmill to perform a symptom limited CPET (Parvo Medics, Sandy, Utah, USA) using a standardized protocol [9, 10]. The heart rate was monitored continuously, with blood pressure and rating of perceived exertion (RPE) obtained at end of each stage. All participants were encouraged to exercise to exhaustion, which was defined as a respiratory exchange ratio (RER) > 1.0. A 10-s averaged sampling interval was used to measure baseline and peak exercise PetCO_2_, ventilatory efficiency (VE/VCO_2_), ventilatory threshold (VT), and oxygen consumption (VO_2_) values. VT was determined by the v-slope method [12] and peak VO_2_ (ml/kg/min) was defined as the maximum oxygen uptake during the last 60 s leading up to exercise termination. Heart rate and blood pressure were monitored continuously throughout the post-exercise scan. Arteriovenous oxygen (A-VO_2_) difference was calculated as previously described [13].

### Cardiovascular magnetic resonance imaging

**Fig. 3:**
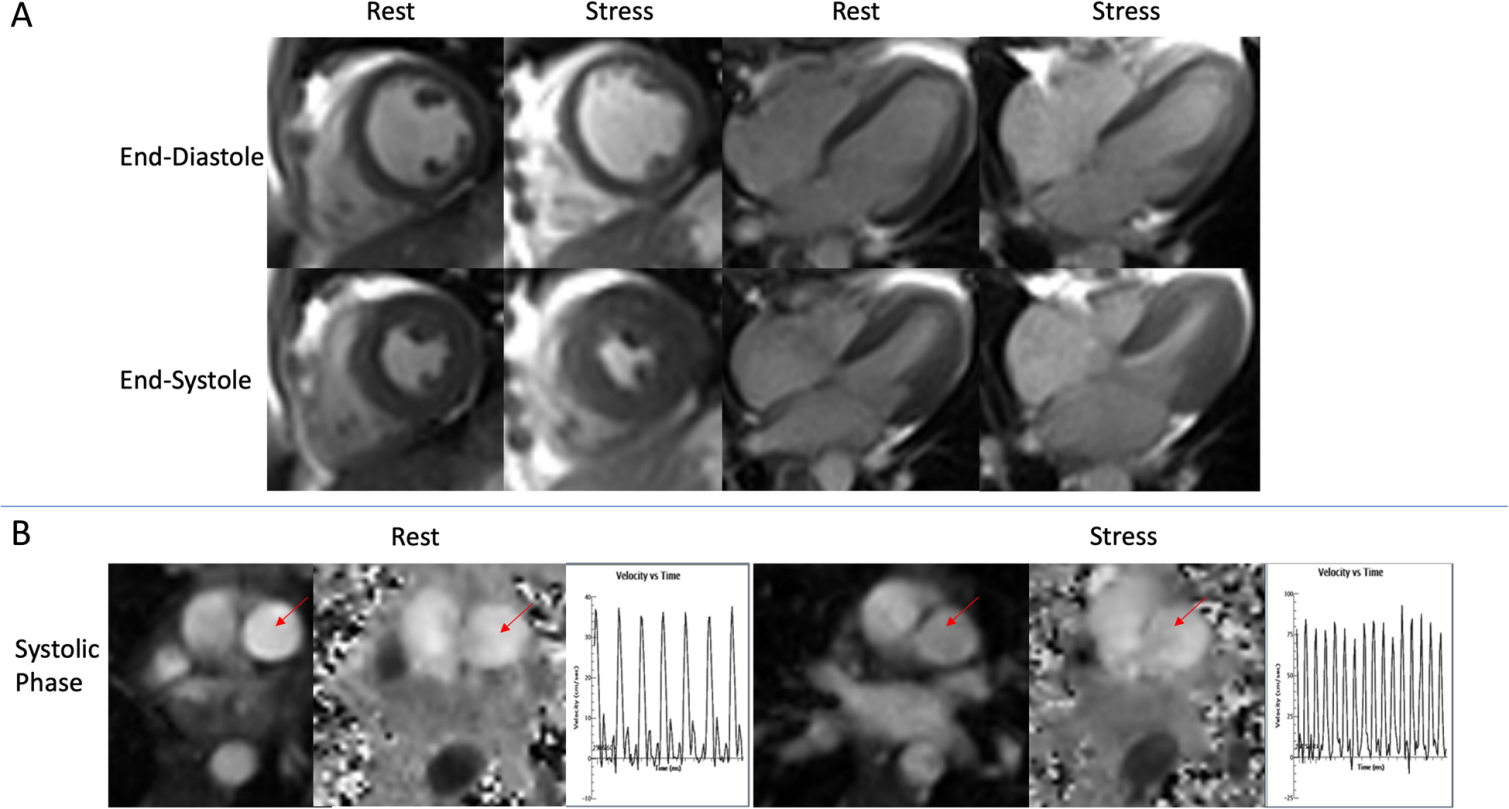
Example pre and peak exercise CMR imagines on a 1.5 T CMR system using a 32-channel phased array coil. **a** Mid short axis slice and HLA for rest and stress images at end-diastole and end-systole. **b** Magnitude and phase images of the main pulmonary artery velocity encoding at rest and stress with readouts. Red arrows pointing to the main pulmonary artery. HLA – horizontal long axis

CMR was performed on a 1.5 T (Espree™, Siemens Healthineers, Erlangen, Germany) using a 32-channel phased array coil and a standard clinical protocol for structural, functional, and phase-contrast breath-hold imaging at rest was followed according to published international guidelines [14]. A real-time, through-plane velocity encoding sequence based on gradient-echo echo-planar imaging (GRE-EPI) with Shared Velocity Encoding (SVE) reconstruction was used for evaluation of PA velocities [15]. An echo train length of 7 and linear k-space acquisition order resulted in an echo time (TE) of 5 ms and a repetition time (TR) of 10 ms at a velocity encoding (VENC) of 150 cm/s. Four shots per image were used to collect a total of 28 k-space lines resulting in an acquisition time of 40 ms for each full k-space dataset [2405 Hz/pixel readout bandwidth, 84 × 128 pixel reconstructed matrix, 10 mm slice, 268 mm × 350 mm rectangular field-of-view (3.2 mm × 2.7 mm pixels)]. A 25° rapid water-excitation pulse was used to suppress fat, and Maxwell correction was used to account for the effect of concomitant gradients on the phase maps. Parallel imaging technique TGRAPPA with acceleration rate 3 was used to reconstruct 84 lines per image. Three orthogonal slices across the PA were prescribed with the highest velocity vector within these slices and the time velocity integral calculated. We used a non-triggered real-time balanced steady-state free precession (bSSFP) acquisition using a radial scheme for k-space acquisition. We chose this method for improved post exercise image quality and reduction of scan time. A total of 60 accelerated short-axis images were acquired for each slice in order to ensure capture of a complete cardiac cycle (approximately 2.5 s of imaging per slice) which allowed for adequate discrimination of ectopic beats. Heart IT software (Heart Imaging Technologies, Durham, North Carolina, USA) allows for manual selection of end-systole and end-diastole for each slice excluding papillary muscles allowing an aggregate compilation of volumes for quantitative ejection fraction. This use of real time cine acquisitions has been utilized previously and shown to have great correlation with standard cine acquisition with less artifact [16]. A stack of real time cine images in the short-axis plane with slice thickness of 8 mm (2-mm inter-slice gap) were acquired fully covering both ventricles from base to apex. For real time cine bSSFP the following parameters were used: TE 0.98 ms; TR 86.26 ms, temporal resolution 46 ms, α = 65°, band width 1488 Hz/pixel. PA velocities, mean and peak gradients were measured at rest and stress See Fig. 3a-e for examples of rest and stress ventricular volumes and pulmonary velocity encoding. Contractile reserve was determined by assessing the change in ejection fraction at peak exercise.

The assessment of RV and LV fibrosis were obtained using late gadolinium enhancement (LGE) imaging 10 min after administration of multihance gadolinium agent (0.15 mmol/kg). Short axis images were acquired using a single shot breath hold phase contrast inversion recovery technique of the entire myocardium. Images were assessed using a semi-quantitive method with HeartIT (Precession) imaging software. This method uses the American Heart Association 17 segment model where six segments for both basal and mid portions, four segments for apical and one segment for apex are scored. Each segment can yield a minimum of 0 for normal myocardium, 1 for 0–25%, 2 for 25–50%, 3 for 50–75% and a maximum of 4 for transmural 75–100% LGE/scar. The sum of the score was used to derive estimated percentage of myocardium with late gadolinium enhancement.

### Statistical analysis

Statistical analysis was performed using SAS (Version 9.4, SAS Institute, Cary, North Carolina, USA). Categorical data were expressed as percentage while continuous variables were reported as mean ± SD along with 95% CI. Two-sided dependent group t-test (α = 0.05) was used to compare the differences between pre and post exercise CMR and CPET observations. To summarize the relationship between ΔCMR parameters and ΔCPET parameters univariate analysis was performed using simple linear regression. Predictors of RVand LV reserve were assessed using stepwise multiple linear regression. *P* values of < 0.05 were considered significant. Bland altman analysis was done in 10 subjects for right and left ventricle ejection fraction to assess for observer variability.

## Results

**Fig. 4:**
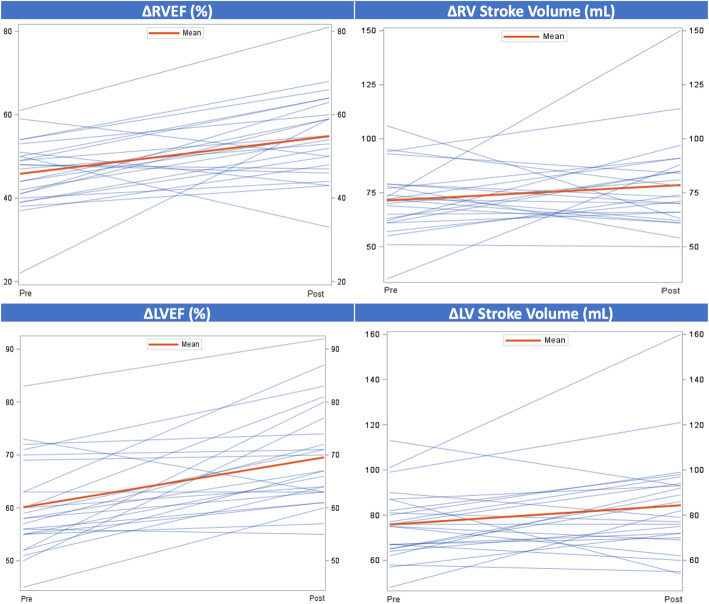
Assessment of RV (Top Panel) and LV (Bottom Panel) contractile reserve. Scatter plots depicting rest and peak changes in ejection fraction and stroke volume for RV (EF *p* = 0.001, SV *p* = 0.08) and LV (EF *p* < 0.001, SV *p* = 0.02). Each line is representative of an individual patient. Dark red line indicates mean value. RV = right ventricle, LV = left ventricle, EF = ejection fraction, SV = stroke volume

**Fig. 5:**
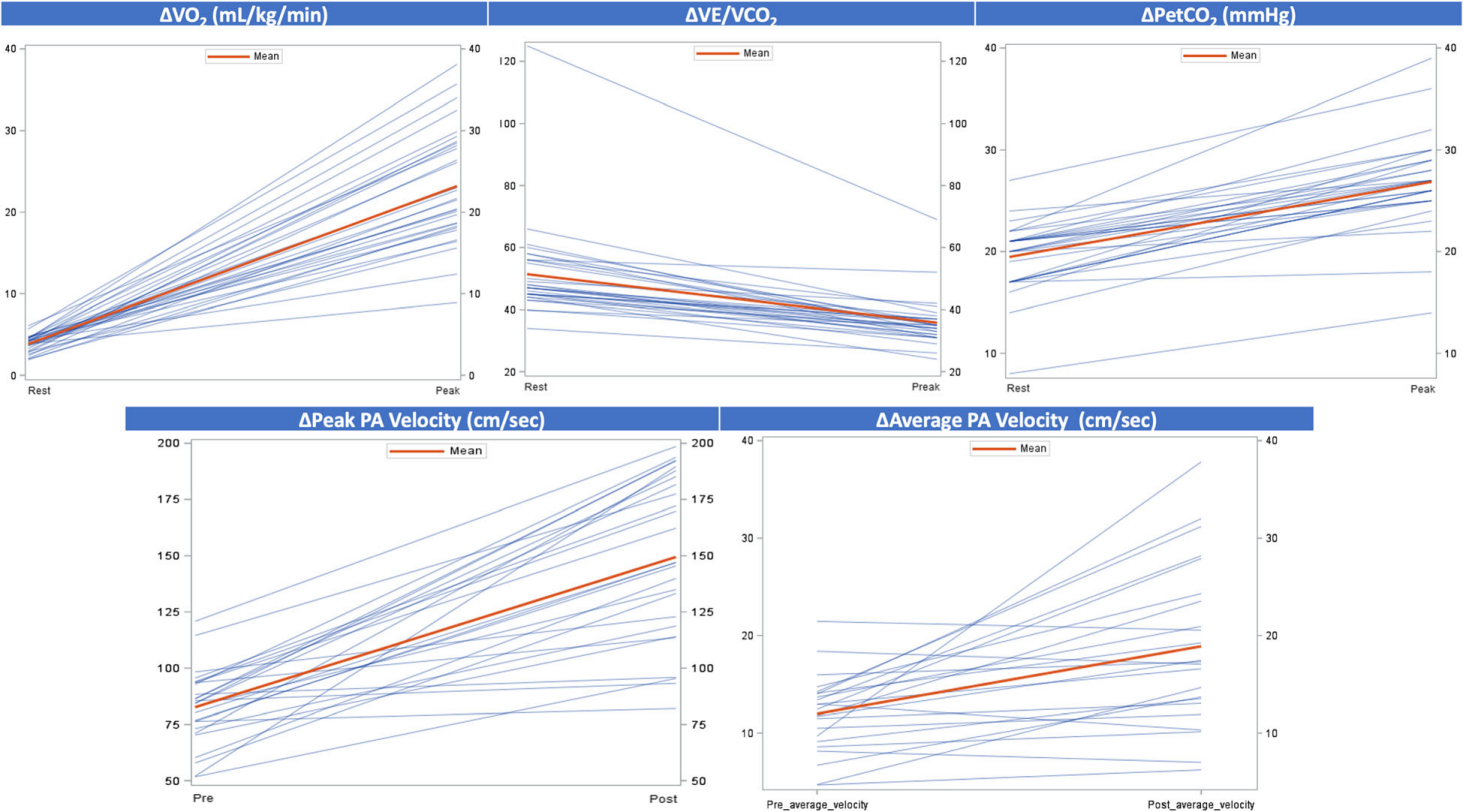
Assessment of cardiopulmonary function and main pulmonary velocities. Top panel indicates resting cardiopulmonary function as assessed with CPET and values measured occur at peak exercise for VO_2_, VE/VCO_2_ and PETCO_2_. Lower panel depicts resting and peak average and peak pulmonary velocities as measured through velocity encoding sequence with VENC set points of 100 for rest and 150 with stress. Each line represents an individual patient. Dark red line indicates mean value

Figure 2 shows subjects undergoing exercise treadmill testing with CPET and CMR analysis. For test subjects, the mean age of the participates was 49.7 ± 11.0 years and the mean body mass index (BMI) was 28.8 ± 7.6 kg/m^2^. The mean duration of HIV exposure was 23.9 years. Table 1 enumerates the baseline characteristics of study cohort, while Table 2 and Table 3 details the specifics of exercise testing. The mean percent of age predicted maximal heart rate was 90.7 ± 17.9 bpm and mean metabolic equivalents (MET)s achieved was 9.6 ± 3.9. The time to transfer the patient, prior to commencing stress imaging, averaged < 35 s, which is within acceptable thresholds advocated by the American Heart Association [17]. During initial screening, pulmonary functional testing (PFTs) was used to assess baseline function. The mean forced expiratory volume in 1 s (FEV_1_) was 2.9 ± 8.6 L and a mean % predicted FEV_1_ of 91.9 ± 18.4 and mean % FEV_1_/ forced vital capacity (FVC) was 92.1 ± 12.0. Maximal voluntary ventilation (MVV), a surrogate for maximal ventilatory capacity, was calculated as FEV_1_ × 40 to measure ventilatory reserve. For the present cohort, the mean MVV was 116 and the mean VE (Max) 67.5 ± 29.2. The ventilatory reserve (VE:MVV) is a measurement of the intrinsic pulmonary limitations to exercise [7] with normative range < 0.7. We found a VE: MVV of 0.6. The pre-exercise imaging was slightly variable, given need for optimization of the phase contrast imaging planes to ensure adequate visualization of the PA and prescription of orthogonal planes for assessment of PA velocities. Figures 4, 5 and Table 4 details the peak exercise hemodynamic parameters. Notably, mean peak VO_2_ was decreased at 23.2 ± 6.9 ml/kg/min and mean VE/VCO_2_ slope was increased at 35.8 ± 7.9 and overall with 20 patients (58.8% of overall cohort) achieving a RER > 1 indicating a maximal exercise effort.

**Table 1:** Demographic characteristics of the EXHALTED population (*n* = 34)

		Total n	Mean ± SD/Frequency (%)	95% CI
Demographics	Age, years	34	49.68 ± 11.01	(45.84-53.52)
	African American	34	28 (82.35)	(68.85-95.85)
	Unemployed	34	26 (76.47)	(61.45-91.49)
	Current smokers	34	20 (58.82)	(41.39-76.25)
	Current alcohol use, yes	34	19 (55.88)	(38.30-73.47)
Labs	Absolute CD4 count	34	699.62 ± 416.81	(554.18-845.05)
	Absolute CD8 count	34	769.59 ± 307.47	(662.31-876.87)
	White Blood Cell count, Thousand/uL	33	5.72 ± 1.97	(5.02-6.42)
	Hemoglobin, g/dl	33	13.59 ± 1.61	(13.02-14.17)
	Creatinine, mg/dL	33	1.02 ± 0.25	(0.93-1.11)
	Albumin, g/dL	33	4.19 ± 0.29	(4.09-4.29)
Medication Use	Aspirin	34	9 (26.47)	(10.85-42.10)
	β-Blockers	34	3 (8.82)	(0.00-18.87)
	Statins	34	9 (26.47)	(10.85-42.10)
	Diuretics	34	2 (5.88)	(0.00-14.22)
	Calcium Channel Blockers	34	6 (17.65)	(4.15-31.15)
	ACE Inhibitors/ARB	34	9 (26.47)	(10.85-42.10)
Medical History	Pulmonary Hypertension, no	33	33 (100.00)	(100.00-100.00)
	Cancer Diagnosis, no	34	30 (88.24)	(76.82-99.65)
	Hypertension, no	34	20 (58.82)	(41.39-76.25)
	Diabetes, no	34	28 (82.35)	(68.85-95.85)
	Hyperlipidemia, no	34	25 (73.53)	(57.90-89.15)
	Coronary artery disease, no	34	33 (97.06)	(91.08-100.00)
	History of stroke, no	34	32 (94.12)	(85.78-100.00)
	Chronic kidney disease, no	34	34 (100.00)	(100.00-100.00)

**Table 2:** Exercise CMR Testing Times

	Mean ± SD	Min	Max
Exercise Time (min:sec)	08:17 ± 03:43	01:21	15:02
Time Exercise End to Start Images (min:sec)	00:34 ± 00:22	00:20	1.11
Pre-exercise Scan (min)*	20-25		
Post-exercise scan (min)*	15-20

**Table 3:** Resting and peak exercise heart and blood pressure

	Mean ± SD	Min	Max
**Resting**			
Heart Rate (bpm)	75.25 ± 14.73	58.00	122.00
Supine Systolic Blood Pressure (mmHg)	122.71 ± 20.22	96.00	173.00
Supine Diastolic Blood Pressure (mmHg)	81.04 ± 12.48	54.00	103.00
**Peak Exercise**			
Heart Rate (bpm)	155.67 ± 36.29	115.00	196.00
% Age Predicted Max Heart Rate	90.7 ± 17.86	67.00	149.00
Systolic Blood Pressure (mmHg)	161.37 ± 20.26	118.00	190.00
Diastolic Blood Pressure (mmHg)	84.37 ± 15.26	52.00	118.00
METS Achieved	9.63 ± 3.89	4.2	16.50

**Table 4:**
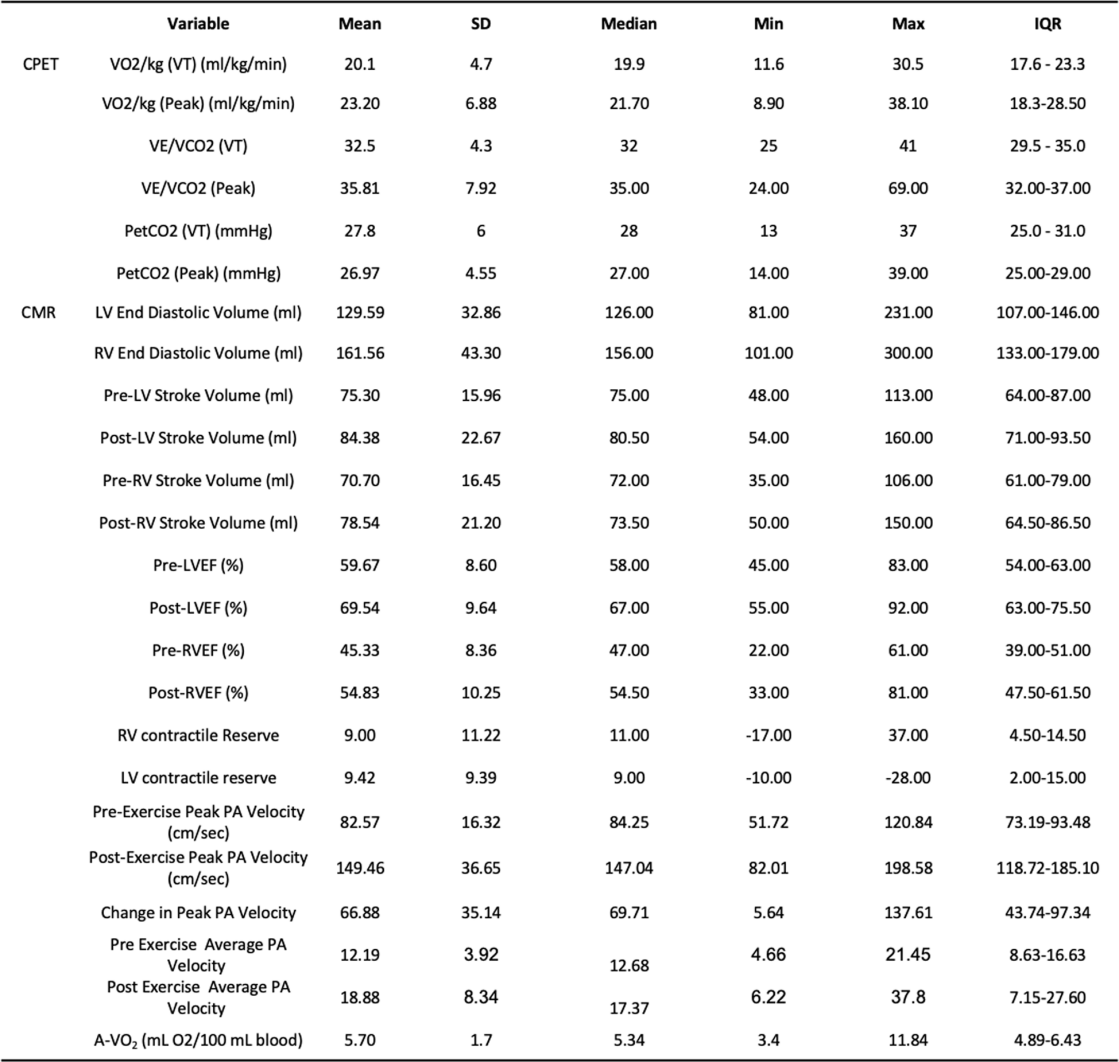
CPET and CMR

### Contractile reserve

To assess contractile reserve, LVEF and RV ejection fraction (RVEF) and stroke volumes were quantified at rest and stress. Basal ventricular function ejection fraction for RV and LV were within normal limits (Fig. 4, Table 4). Using an absolute increase of 5% as a measure of contractile reserve [4], both RV and LV augmented appropriately with exercise (Fig. 4, Table 4). The mean absolute contractile reserve for RV was 9.0% ± 11.2 and LV was 9.4% ± 9.4. The mean change in RV and LV stroke volume was 7.2 ± 25.3 ml and 8.6 ± 19.7 ml, respectively. We measured peak and average PA velocities as well as mean and peak gradients for rest and stress to evaluate for evidence for PAH. We have previously shown exceptional correlation between PA velocities, namely average PA velocity with invasively measured mean PA pressures and calculated pulmonary vascular resistance (PVR) with right heart catheterization at rest in PAH patients [18]. Elevated mean PA pressures correlated with a reduced average PA velocity as derived from CMR. The mean average PA velocity for rest and stress was 12.2 ± 3.9 and 18.9 ± 8.3 (See Fig. 5, Table 4) indicating the absence of exercise-induced PAH. The mean and peak gradients did not change significantly with exercise and remained within normal limits (Data not shown).

### Metabolic gas exchange relationships

We further assessed the relationship between functional changes in LVEF, RVEF and peak PA velocity relative to gas exchange parameters. Correlation matrix analysis between change in VO_2,_ VE/VCO_2_, PetCO_2_ relative to the change in RVEF, stroke volume and PA velocities (Supplemental Table 1). The change in LVEF, RVEF, peak or average PA velocity did not account for changes in VO_2_, peak PetCO_2_ and VE/VO_2_. Interestingly, a relationship was observed between change in PetCO_2_ in response to exercise and change in LV and RV stroke volume (Supplemental table 1).

### Assessment of skeletal muscle strength

Prior to exercise testing, patients baseline skeletal muscle strength was assessed by hand grip (Lafayette Instrument, Lafayette, Indiana, USA) and quadricep (Hoggan Scientific, LLC, Salt Lake City, Utah, USA) strength. Participants were graded on a scale of 0–5. Scores were averaged over 3 trials. We did not find significant evidence of muscle weakness in hand and quadriceps testing as all patients scored greater than 4 with testing.

### Assessment of LV and RV fibrosis

The presence of scar/fibrosis portends a poor prognosis in many cardiomyopathies. To assess for scar/fibrosis, we performed LGE imaging using single shot breath hold inversion recovery sequences in addition to stress imaging protocols. The presence of RV enhancement was not identified; however enhancement was seen in the LV in five (15%) of 34 patients with average scar burden of 5.6%. Predominant location of scar was found in the mid myocardium or junctional RV insertion site. Subendocardial or transmural scar indicative of prior infarct was not observed.

For consistency, CMR images were read by two experienced cardiologists for RV and LV volumetric analysis as well as average velocities. We used random effects model to evaluate inter-rater reliability [19]. Our results show appropriate reliability for RV volumetric analysis with ICC = 0.73 and LV volumetric analysis with ICC = 0.82. Additionally, we found agreement for average PA velocity at rest with ICC = 0.70 and stress ICC = 0.72. See supplemental figure 1 & 2.

## Discussion

In the present study, we sought to evaluate causes of dyspnea in a cohort of well treated HIV patients with exercise limitation and no overt signs of PAH using a comprehensive combined CPET/CMR platform that would allow unprecedented ability to simultaneously assess for multiple central and peripheral etiologies of dyspnea [7]. Overt evidence of lung disease and preexisting cardiac dysfunction and resting pulmonary hypertension were excluded through screening procedures such as chest CT, echocardiogram and PFT’s. Parenchymal lung disease, abnormalities in resting ventilatory reserve and reactive airway disease were excluded. VE:MVV, a measurement of the intrinsic pulmonary limitation to exercise [7] was within normal limits (< 0.7), with mean VE:MVV at 0.6, which made intrinsic lung pathology less likely. The feasibility of combined exercise CPET and CMR has been previously noted by other groups for investigation of cardiopulmonary pathology [13, 20]. The juxtaposition of these technologies greatly simplifies the assessment of the patient with unexplained dyspnea and facilitates diagnosis through an iterative process of exclusion. We ensured that patients were exercised to their peak capacity, with transfers occurring rapidly, ensuring that imaging was performed within suggested parameters for near peak exercise imaging of the ventricles [17].

We initially identified substantially impaired maximal exercise capacity (VO_2_ peak and VCO_2_) in this HIV patient population with mild subjective dyspnea. This is well described and has been noted by us and others [21, 22]. Given the impairment of peak VO_2_ capacity, we systemically interrogated for cardiac causes of dyspnea. We considered exercise-induced increase in pulmonary vascular resistance, given that this may present with dyspnea and considered an early marker for resting PAH [8, 23, 24]. This diagnosis could be very easily ascertained through assessment of pulmonary artery velocities with real-time phase contrast CMR assessment of PA velocities at peak exercise. The mean average PA velocities in this population at rest and with exercise corresponded to a normal range of mean PA pressures, using previously described regression equations that have correlated mean average PA velocities with invasive right heart catheterization in patients with PAH [18]. We were able to image the PA reproducibly to obtain a reliable estimate for PA velocities at peak exercise in all patients.

Poor contractile reserve may also help explain dyspnea in patients with otherwise structurally normal resting ventricles but has not been adequately evaluated in HIV patients with dyspnea. The presence of contractile reserve in patients with nonischemic cardiomyopathy, irrespective of stressors and imaging modality is associated with 80% reduction in mortality and hospitalization [4]. RV contractile reserve is particularly important in patients with the diagnosis of PAH, where inadequate ventricular response to exercise may predict eventual development of fixed PAH and mortality [6, 8]. We did not find evidence of compromised biventricular contractile reserve. We then turned our attention to myocardial scar/fibrotic burden as early indications of dysfunction and found only a minority of subjects (5 patients) could be identified with LGE which primarily involved the LV and not the RV. There are reports of higher predisposition for fibrosis in HIV patients compared to age match control populations [25]. In patients with HIV, LGE at the RV insertion site, inferolateral wall or septum has been previously noted and thought to be prevalent and related to prior myocarditis [26, 27]. However, we did not consistently observe LGE, suggesting a lack of RV/LV remodeling to fully explain symptomology. We measured diastolic indices in the LV and RV at rest and with exercise and did not observe any changes in diastolic function excluding a contribution of diastolic dysfunction (data not shown).

In the setting of mean RER 1.0, we observed reduced peak VO_2_ (23.2 ml/kg/min) and elevated peak VE/VCO_2_ (mean 35.8). The mean PetCO_2_ at VT was also reduced at 27 mmHg. Lewis et al., demonstrated in patients with heart failure with reduced ejection fraction, VE/VCO_2_ slope is negatively correlated to RVEF and significantly associated with pulmonary vascular resistance during exercise, without relationship to LVEF or systemic resistance [28]. These changes were ameliorated by the addition of a selective inhibitor of type 5 phosphodiesterase [28]. In our observations, there was no relationship between VE/VCO_2_ slope and resting RVEF or with RV contractile reserve. This may have been related to the fact that our patient population did not have evidence of resting or provoked abnormalities in contractile function. However, the variability of LV and RV stroke volume were directly related to changes in PetCO_2_ (Supplemental Table 1). Previous studies have found a relationship between PetCO_2_ values and cardiac output as well as severity of heart failure [29, 30], however the significance of this finding is unclear and may warrant further investigation in future studies. The finding of VE: MVV of 60%, demonstrated a normal breathing reserve, normal PFTs and lack of elevated right sided pressures suggest that a pulmonary etiology is unlikely, and that dyspnea may relate to peripheral mechanisms of alterations in VO_2_ peak.

Skeletal muscle mass is an independent predictor of peak VO_2_ and VE/VCO_2_ slope, which could explain our observations [31]. Peripheral limitations to exertion in people living with HIV can include sarcopenia [32], skeletal myopathy from direct mitochondrial toxicity [26], and prolonged exposure to elevated systemic inflammation [33]. Skeletal myopathy in patients with HIV can occur as a result of direct/prolong exposure to the virus. Additionally, anti-retroviral therapies may cause myopathies through direct mitochondrial toxicity [34]. Although macroscopic muscle strength testing did not show overt evidence of a manifest myopathy, a subclinical process could help explain dyspnea in the patient cohort. The average duration of HIV exposure in our patient population was 23.9 years, which likely indicates prolong exposure to the virus and anti-retroviral therapies. Although majority of patients reached a RER of 1, there were a notable number of patients unable to reach target exertion goals suggesting either deconditioning or symptom limiting fatigue. Indeed, subjective dyspnea may be a reflection of overall deconditioning and may not be related to progression of disease. The average BMI was 29 Kg/m^2^, suggesting that majority of patients were overweight, if not obese. Indeed, there is a rising epidemic of overweight/obesity amongst HIV patients which will have implications for cardiovascular health [35].

Prior studies have used comparable system with CMR compatible ergometer/treadmills coupled with CPET in pediatric patients with PAH and tetralogy of Fallot [12]. Although similar, our system utilized a CMR compatible hydraulic treadmill with gradual stress protocols [10, 17]. The advantage of using a combined CMR/CPET platform is the ability to comprehensively assess cardiopulmonary function with ventilatory function, quantification of RV/LV volumes, velocity encoding of great vessels and scar/fibrosis burden with LGE and evaluation of electrocardiogram findings. Conditions such as low flow/low gradient aortic stenosis, load dependent mitral valve regurgitation and monitoring of treatment response of patients with PAH are amongst emerging areas of interest for comprehensive cardiopulmonary evaluation.

### Limitation

A major limitation of this study was that it was conducted in a single center small cohort of HIV patients. The small sample size reflects the challenge in recruiting patients who met the specific study requirements of not having evidence of structural disease or baseline contractile dysfunction but were found to be symptomatic while meeting the additional criteria of undergoing exercise testing in a the CMR environment. We utilized average velocity with CMR derived velocity encoding sequence across the main PA as a surrogate for mean PA pressure. Although previous studies have shown acceptable correlation between average resting PA velocity and invasively measured mean PA velocity it is unknown if the average velocity at peak stress correlates closely with invasively measured mean PA pressure under exercise conditions. An additional limitation of the study is we did not measure peripheral lean muscle mass to screen for sarcopenia as a correlate of submaximal exercise performance.

## Conclusion

Using treadmill stress with CMR/CPET analysis provided comprehensive cardiopulmonary function testing. The data suggests that in well treated patients with HIV, contractile dysfunction with exercise, exercise PAH, diastolic function or presence of the RV/LV scar are not significant contributors to unexplained dyspnea. Replication of this data in a large multicenter approach may increase the clinical applicability. The etiology of symptoms points to a non-cardiopulmonary etiology that may involve subclinical myopathy related to chronic exposure to the HIV virus, drugs and/or physical deconditioning. Future studies may evaluate poorly or uncontrolled retroviral illness in assessing cardiopulmonary function with comprehensive platforms such as CMR/CPET.

## Supplementary information

**Additional file 1 Figure S1** Bland Altman analysis of right ventricular ejection fraction (RVEF) and left ventricular ejection fraction (LVEF) contractile reserve.

**Additional file 2: Figure S2** Bland Altman analysis of rest and stress average pulmonary artery velocity as assessed using real time velocity encoding sequence.

**Additional file 3 Table S1** Pearson Correlation Coefficients

## Data Availability

The datasets used and/or analyzed during the current study are available from the corresponding author on reasonable request.

## Abbreviations

A-VO2: Arteriovenous oxygen difference
BMI: Body mass index
bSSFP: Balanced steady state free precession
CMR: Cardiovascular magnetic resonance
CPET: Cardiopulmonary exercise testing
CT: Computed tomography
eGFR: Estimated glomerular filtration rate
EPI: Echo planar imaging
FEV_1_: Forced expiratory volume in one second
FVC: Forced vital capacity
GRE: Gradient echo
HIV: Human immunodeficiency virus
LGE: Late gadolinium enhancement
LV: Left ventricle/left ventricular
LVEF: Left ventricular ejection fraction
METs: Metabolic equivalents
MMRC: Modified Medical Research Council
MVV: Maximal voluntary ventilation
PA: Pulmonary artery
PAH: Pulmonary artery hypertension
PFT: Pulmonary function test
PVR: Pulmonary vascular resistance
RER: Respiratory exchange ratio
RPE: Rating of perceived exertion
RV: Right ventricle/right ventricular
RVEF: Right ventricular ejection fraction
SV: Stroke volume
SVE: Shared velocity encoding
TE: Echo time
TR: Repetition time
VE: Ventilatory efficiency
VENC: Velocity encoded
VO_2_: Oxygen consumption
VT: Ventilatory threshold
