# Supplementary material for: Evaluation of dyspnea of unknown etiology in HIV patients with cardiopulmonary exercise testing and cardiovascular magnetic resonance imaging
Source: J Cardiovasc Magn Reson. 2020 Oct 12;22:74. doi: 10.1186/s12968-020-00664-6 (PMC7549205; doi:10.1186/s12968-020-00664-6)
Supplement: Supplementary file 1 — Additional file 1 Figure S1 Bland Altman analysis of right ventricular ejection fraction (RVEF) and left ventricular ejection fraction (LVEF) contractile reserve. [file 12968_2020_664_MOESM1_ESM.docx]

**Supplemental Figure 1.**

Bland Altman analysis of right ventricular ejection fraction (RVEF) and left ventricular ejection fraction (LVEF) contractile reserve.
