# Supplementary material for: Evaluation of dyspnea of unknown etiology in HIV patients with cardiopulmonary exercise testing and cardiovascular magnetic resonance imaging
Source: J Cardiovasc Magn Reson. 2020 Oct 12;22:74. doi: 10.1186/s12968-020-00664-6 (PMC7549205; doi:10.1186/s12968-020-00664-6)
Supplement: Supplementary file 2 [file 12968_2020_664_MOESM3_ESM.docx]

Supplemental Table 1. Pearson Correlation Coefficients

	Δ LVEF	Δ RVEF	Δ LV Stroke Volume	Δ RV Stroke Volume	Δ Peak Velocity	Δ Average PA Velocity	Δ VO2kg	Δ PetCO2	Δ VE/VCO2
Δ LVEF	1								
	24								
Δ RVEF	0.73	1							
	<.0001								
	24	24							
Δ LV Stroke Volume	0.50	0.38	1						
	0.01	0.07							
	24	24	24						
Δ RV Stroke Volume	0.51	0.52	0.91	1					
	0.01	0.01	<.001						
	24	24	24	24					
Δ Peak Velocity	-0.07	-0.07	0.01	-0.07	1				
	0.73	0.74	0.98	0.76					
	24	24	24	24	26				
Δ Average PA Velocity	0.06	0.21	-0.24	-0.20	0.14	1			
	0.79	0.34	0.29	0.36	0.54				
	22	22	22	22	23	23			
Δ VO2kg	0.15	0.07	-0.36	-0.35	0.02	0.28	1		
	0.50	0.75	0.10	0.10	0.92	0.20			
	23	23	23	23	25	23	31		
Δ PetCO2	0.36	0.01	0.48	0.46	0.08	0.02	0.08	1	
	0.10	0.97	0.02	0.03	0.73	0.92	0.68		
	22	22	22	22	24	22	30	30	
Δ VE/VCO2	-0.30	0.02	-0.30	-0.25	-0.10	0.02	0.03	-0.47	1
	0.16	0.94	0.16	0.25	0.62	0.94	0.88	0.01	
	23	23	23	23	25	23	31	30	31
